# Epidemiology and seasonality of human parainfluenza serotypes 1‐3 in Australian children

**DOI:** 10.1111/irv.12838

**Published:** 2021-01-24

**Authors:** Daniel R. L. Greiff, Alice Patterson‐Robert, Christopher C. Blyth, Kathryn Glass, Hannah C. Moore

**Affiliations:** ^1^ Wesfarmers Centre for Vaccine and Infectious Diseases Telethon Kids Institute University of Western Australia Perth WA Australia; ^2^ Medical School College of Health and Medicine Australian National University Canberra ACT Australia; ^3^ School of Medicine University of Western Australia Perth WA Australia; ^4^ Department of Infectious Diseases Perth Children’s Hospital Perth WA Australia; ^5^ Department of Microbiology PathWest Laboratory Medicine Perth WA Australia; ^6^ Research School of Population Health Australian National University Canberra ACT Australia

**Keywords:** data linkage, hospitalisation, incidence, infants, parainfluenza viruses, seasonality

## Abstract

**Background:**

Parainfluenza viruses are significant contributors to childhood respiratory illness worldwide, although detailed epidemiological studies are lacking. Few recent Australian studies have investigated serotype‐specific PIV epidemiology, and there is a paucity of southern hemisphere PIV reports. We report age‐stratified PIV hospitalisation rates and a mathematical model of PIV seasonality and dynamics in Western Australia (WA).

**Methods:**

We used linked perinatal, hospital admission and laboratory diagnostic data of 469 589 children born in WA between 1996 and 2012. Age‐specific rates of viral testing and PIV detection in hospitalised children were determined using person time‐at‐risk analysis. PIV seasonality was modelled using a compartmental SEIRS model and complex demodulation methods.

**Results:**

From 2000 to 2012, 9% (n = 43 627) of hospitalised children underwent PIV testing, of which 5% (n = 2218) were positive for PIV‐1, 2 or 3. The highest incidence was in children aged 1‐5 months (PIV‐1:62.6 per 100 000 child‐years, PIV‐2:26.3/100 000, PIV‐3:256/100 000), and hospitalisation rates were three times higher for Aboriginal children compared with non‐Aboriginal children overall (IRR: 2.93). PIV‐1 peaked in the autumn of even‐numbered years, and PIV‐3 annually in the spring, whereas PIV‐2 had inconsistent peak timing. Fitting models to the higher incidence serotypes estimated reproduction numbers of 1.24 (PIV‐1) and 1.72 (PIV‐3).

**Conclusion:**

PIV‐1 and 3 are significant contributors towards infant respiratory hospitalisations. Interventions should prioritise children in the first 6 months of life, with respect to the observed autumn PIV‐1 and spring PIV‐3 activity peaks. Continued surveillance of all serotypes and investigation into PIV‐1 and 3 interventions should be prioritised.

## 1 INTRODUCTION

Acute lower respiratory infections (ALRIs) are an important cause of childhood hospitalisation globally. Alongside respiratory syncytial virus (RSV) and influenza viruses, human parainfluenza viruses (PIVs) are increasingly understood to contribute greatly to paediatric respiratory tract infections. Though PIV‐associated mortality is low in high‐income countries, morbidity remains high. PIVs are the second most common cause of childhood ALRI‐associated hospitalisation, after RSV; comparable in frequency to influenza.^1^, ^2^ PIVs were found to account for 6.8% of all hospitalisations for fever, ALRI or both, in children aged less than 5 years in the United States between 2000 and 2004.^3^ Additionally, the healthcare costs associated with PIV hospitalisation in the United States were estimated to be over $250 million USD annually between 1998 and 2010.^4^

PIVs are a group of four serologically distinct human viruses, numbered 1‐4, of the *Paramyxoviridae* family.^5^ PIVs can cause a variety of respiratory infections, from mild upper respiratory illnesses in healthy adults, to croup, bronchiolitis, and pneumonia in infants, children, and severe lower respiratory infection in the elderly, and the immunocompromised.^6^ The four serotypes are known to manifest distinctively, with PIV‐1 and 2 being the most common aetiologic agents of croup, whereas PIV‐3 and 4 are more commonly associated with bronchiolitis and pneumonia.^6^, ^7^ There are no licensed prophylactic agents, vaccinations or therapies for any PIV serotype^8^, ^9^, ^10^; development having stalled in part due to the paucity of studies on their health burden.^11^

Infants and children are a particular risk group for infection with PIVs, and all serotypes are known to result in hospitalisation in young children.^12^, ^13^, ^14^, ^15^ Though all serotypes are common causes of childhood illness, PIV‐3 and 1 are thought to be the most frequent causes of hospitalisation, with PIV‐2 presenting a slightly lower health burden.^12^, ^13^, ^14^ The epidemiology of PIVs in resource‐poor settings remains poorly understood, particularly in the tropical and subtropical regions of the southern hemisphere, despite the high rates of ALRI‐associated mortality which persist in these environments.^16^ Studies in America and Japan have reported spring peaks in PIV‐3 circulation, and autumn and winter peaks for PIV‐1 and 2 across the northern hemisphere.^12^, ^13^, ^14^, ^15^ Although biennial peaks in PIV‐1 activity, generally in odd‐numbered years, are usually reported, one Korean study observed relatively indistinct PIV‐1 peaks.^17^ There is even less consensus on yearly PIV‐2 fluctuations, with some studies reporting biennial, others reporting annual and others reporting only sporadic outbreak‐like activity.^4^, ^12^, ^15^, ^17^ One Brazilian study identified biannual PIV‐2 peaks, which have not been observed elsewhere to date.^18^ Overall, reports on PIV epidemiology from outside temperate, northern hemisphere regions remain rare: the most comprehensive existing reviews of respiratory virus seasonality often lack PIV serotype specificity.^2^, ^19^

This study set out to investigate the serotype‐specific epidemiology of PIV‐1 and 3 in Western Australian children, reporting incidence rates of laboratory‐confirmed PIV hospitalisation by age group and Aboriginal status, and seasonal parameters through dynamic transmission models.

## 2 METHODS

### 2.1 Study setting

Western Australia (WA) encompasses the western third of Australia, an area of approximately 2.5 million square kilometres, and, as of 2013, contained approximately 2.5 million people, around 100 000 of whom identify as being of Aboriginal and/or Torres Strait Islander origin (hereafter respectfully referred to as Aboriginal). Three quarters of the population reside in the temperate climatic region of metropolitan Perth and its surrounds.^20^

### 2.2 Data sources and study cohort

**FIGURE 1:**
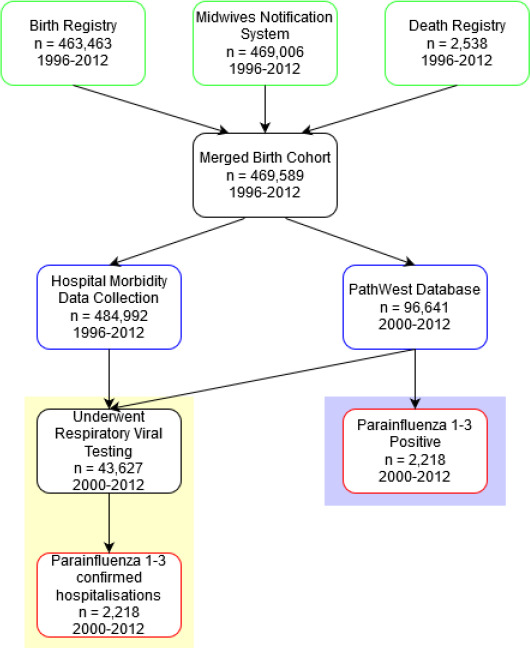
Process of dataset construction and record linkage. Databases used in birth cohort construction are in green boxes, and administrative hospital and laboratory databases are in blue boxes. Note that the number of admissions differs from the size of the birth cohort, as multiple admissions per person are permitted. The yellow‐shaded box highlights the analytic dataset used for incidence rate calculation, while the purple‐shaded box highlights analytic dataset for seasonality and transmission models

This analysis formed part of a larger study investigating the pathogen‐specific epidemiology of respiratory infections in a population‐cohort of WA births from 1996 to 2012 using probabilistically linked administrative data. Full study details are available elsewhere.^1^ In brief, we identified a cohort of 469 589 WA‐born infants with unit‐record linked data from WA’s state‐wide Birth and Death Registry, Midwives Notification System, Hospital Morbidity Data Collection (HMDC), and PathWest Laboratory Medicine databases (Figure 1). Aboriginal children were identified using a validated algorithm across multiple datasets.^21^ Probabilistic data linkage was conducted by the WA Health Data Linkage Branch using best practice protocols.^22^, ^23^

### 2.3 Parainfluenza virus episodes

PathWest is WA’s leading public pathology provider, and, as the state's major referral pathology laboratory, receives specimens from all public and private hospitals. Respiratory samples collected routinely from children aged 16 and under, mostly in hospitals, underwent routine viral diagnostic panels involving serology, culture, direct antigen detection and polymerase chain reaction (PCR) testing. Positive and negative results for PIV‐1, 2 and 3 were available. The WA HMDC is a state‐wide dataset recording admissions to private and public hospitals, psychiatric hospitals, and day surgeries across WA.^23^ Hospital admissions were linked to laboratory diagnostic records for specimens collected from the same individual within 48 hours of the hospital admission date.^1^ We restricted hospital admission records to the period covering 2000‐2012, as laboratory records were only available from 2000.

### 2.4 Statistical analysis

We used two different primary outcome measures for this study. The first of these, used to calculate incidence rates, was hospitalisations in children between 2000 and 2012 with a positive detection of PIV‐1, 2 or 3 from a respiratory specimen using any of the routine diagnostic approaches. As PIV laboratory confirmation is not conducted in all hospitalised children, we also assessed the rate of testing. The secondary outcome measure was all positive detections of PIV‐1, 2 and 3, regardless of hospitalisation status. This measure was used to investigate seasonal dynamics using complex demodulation and dynamic transmission models.

#### 2.4.1 Incidence rates

The incidence rates of PIV‐1, 2 and 3 were calculated using time to event survival analysis methods allowing for multiple PIV hospitalisations per person, as in previous analyses of this dataset for RSV.^24^ Person time‐at‐risk was calculated from the start of the study period (1st January 2000, or from the date of birth) until the time of study exit, taken as either the 31st of December 2012, the date of death, or date of hospitalisation for PIV or for respiratory viral testing. We calculated incidence rates of testing and PIV‐confirmed hospitalisations with 95% confidence intervals (95% CI) according to age and Aboriginal status. Analyses were conducted using STATA SE (version 14.1) by Stata Corp.

#### 2.4.2 Seasonality

To assess seasonality, we used data on all PIV laboratory detections for children in the birth cohort as we had no reason to believe the seasonality would differ between hospitalised and non‐hospitalised children. SEIR compartmental models are a useful tool for modelling infectious diseases and, when fitted to incidence data, can inform on transmission parameters and seasonality. SEIR models categorise all individuals in a population as either susceptible (S), infected but not yet infectious (E), infectious (I) or recovered (R). We modelled transmission of each serotype of PIV using a deterministic SEIRS model with a single age class, waning immunity and seasonality in transmission (Table S1). The models were implemented and fitted in MATLAB using the ode45 differential equation solver and fitted to data using fminsearch.

Complex demodulation is an analytic approach for cyclical or seasonal time series data that extract the timing and size of peaks over time, and has been used to analyse sleep cycles, cardiovascular variability, and suicides, in addition to seasonal pathogens such as RSV.^25^, ^26^, ^27^, ^28^ Full details of this method are provided elsewhere.^29^ Briefly, the method deconstructs the time series into an amplitude and phase, assuming a given periodic frequency. We applied this approach to weekly PIV incidence counts for serotypes 1‐3 individually, assuming a 52‐week period and using a 52‐week moving average filter.

### 2.5 Ethics

Approvals for this study were obtained from the WA Department of Human Research Ethics Committee and the WA Aboriginal Health Ethics Committee. Data access was provided by the WA Data Linkage Branch.

## 3 RESULTS

### 3.1 PIV incidence rates

**FIGURE 2:**
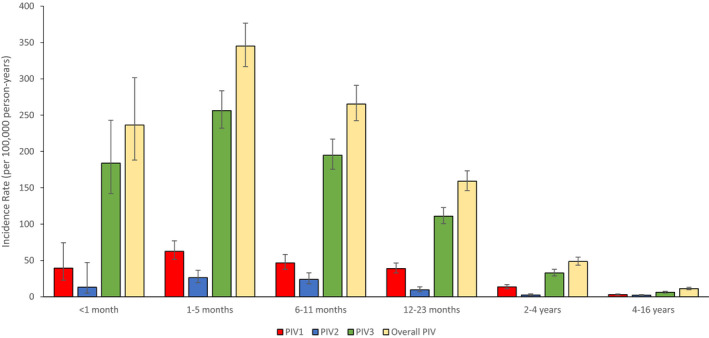
Overall PIV‐1 and 3 hospitalisation rates by age strata for all children aged less than 16 years, Western Australia 2000‐2012. All rates presented per 100 000 person‐years, with error bars representing 95% confidence intervals

From 2000 to 2012, 484 992 hospital admissions were recorded, and 43 627 respiratory viral tests were performed (Figure 1). Of hospitalisations with a linked laboratory record, a combined 2218 were positive for PIV: 487 for PIV‐1, 183 for PIV‐2, and 1548 for PIV‐3. Table 1 shows the frequency of PIV testing in hospitalisations in Aboriginal and non‐Aboriginal children. Hospitalisations that underwent testing for PIV were 1.7‐3.3 times higher in Aboriginal children than non‐Aboriginal children with the per cent positivity ranging from 2% in neonates (aged less than 1 month) to 6%‐7% in children aged 12‐23 months (Table 1). Immunofluorescence was the most common diagnostic method used to detect PIV (52.1% of laboratory‐linked hospitalisations), with 23% being detected by PCR. Croup was the most common primary diagnosis for PIV‐1 (28.5%) and PIV‐2 (29.5%) confirmed hospitalisations, whereas bronchiolitis was the most common primary diagnosis for PIV‐3 (28.5%). Overall, 74% of all PIV‐1 and 3 infections occurred in children aged less than 2 years, and PIV‐3 had the lowest median age of infection (11 months), followed by PIV‐2 (12 months) and PIV‐1 (15 months). For each PIV serotype, incidence rates peaked in children aged from 1 to 5 months (Table S2). The highest hospitalisation rate overall was observed for PIV‐3 in this age group (256 per 1000 000 child‐years, 95% CI: 232‐284), followed by PIV‐1, at 63 per 100 000 (95% CI: 51.4‐76.9; Figure 2). PIV‐2 had the lowest of all incidence rates observed, peaking at 26 per 100 000 child‐years. Incidence rates remained elevated for the first year of life, before declining at 12‐23 months, and again in children aged 4‐16 years. Compared to non‐Aboriginal children, Aboriginal children had approximately three times the overall rate of PIV detection across all age groups (IRR: 2.93, 95% CI: 2.62‐3.27; Table S2). The rate of PIV‐3 in Aboriginal children aged 1 to 5 months was 829 per 100 000 child‐years (95% CI: 669‐1000), almost four times the rate observed in non‐Aboriginal children of that age group (IRR: 3.85, 95% CI: 2.99‐4.92), and PIV‐1 and 2 were both similarly found to have a higher incidence rate in Aboriginal children. The rate of PIV hospitalisation remained higher for Aboriginal children aged 6 to 11 months for PIV‐1 (IRR: 4.35, 95% CI: 2.49, 7.29), PIV‐2 (4.79, 95% CI: 2.18, 9.75) and PIV‐3 (IRR: 3.91, 95% CI: 3.00, 5.06).

**TABLE 1:** PIV‐1 and 3 testing rates by age group and Aboriginal status in Western Australia, 2000‐2012

Age group	Aboriginal			Non‐aboriginal			Incidence rate ratio (95% CI)
	N tested	N positive (% positive)	Testing rate (95% CI)	N tested	N positive (% positive)	Testing rate (95% CI)	
<1 mo	352	6 (1.70%)	173 (156, 193)	2919	66 (2.26%)	103 (99.0, 107)	1.69 (1.51, 1.89)
1‐5 mo	1983	110 (5.50%)	196 (187, 205)	9278	414 (4.46%)	65.5 (64.2, 66.8)	2.99 (2.85, 3.14)
6‐11 mo	1478	108 (7.30%)	123 (116, 129)	6304	370 (5.87%)	37.5 (36.6, 38.4)	3.27 (3.08, 3.46)
12‐23 mo	1200	89 (7.42%)	50.1 (47.3, 53.0)	7806	477 (6.11%)	23.6 (23.0, 24.1)	2.13 (2.00, 2.26)
2‐3 y	733	41 (5.59%)	15.6 (14.5, 16.8)	5631	296 (5.26%)	8.73 (8.50, 8.96)	1.79 (1.65, 1.93)
4‐16 y	736	34 (4.62%)	5.08 (4.72, 5.46)	5206	207 (3.98%)	2.61 (2.54, 2.68)	1.95 (1.80, 2.11)
Total	6482	388 (5.99%)	27.01 (26.3, 27.7)	37 144	1830 (4.93%)	11.2 (11.1, 11.3)	2.41 (2.34, 2.47)

Note

All rates presented per 100 000 person‐years.

### 3.2 PIV seasonality

**FIGURE 3:**
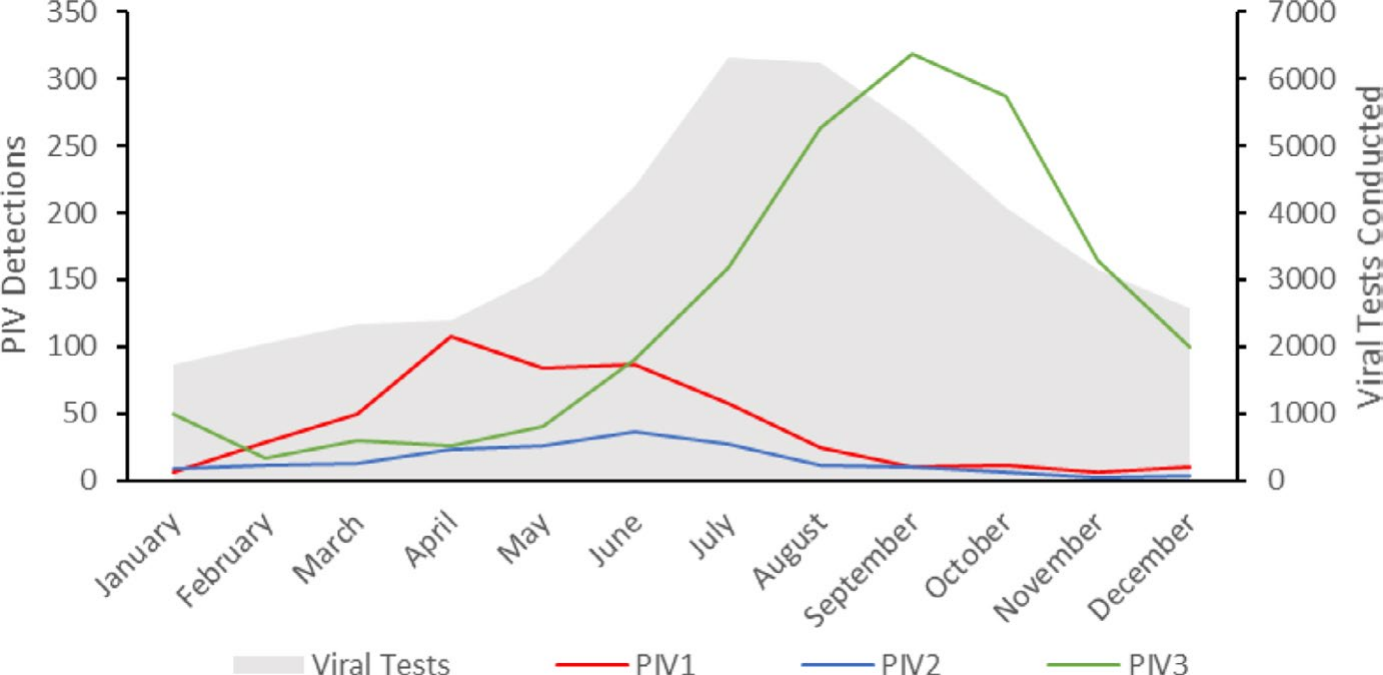
Overall seasonal distribution of PIV‐confirmed hospitalisations in Western Australia, 2000‐2012. Cases from all age groups were tabulated for each month and plotted. Note the right‐side axis, corresponding to monthly viral testing counts (shaded graph area)

**FIGURE 4:**
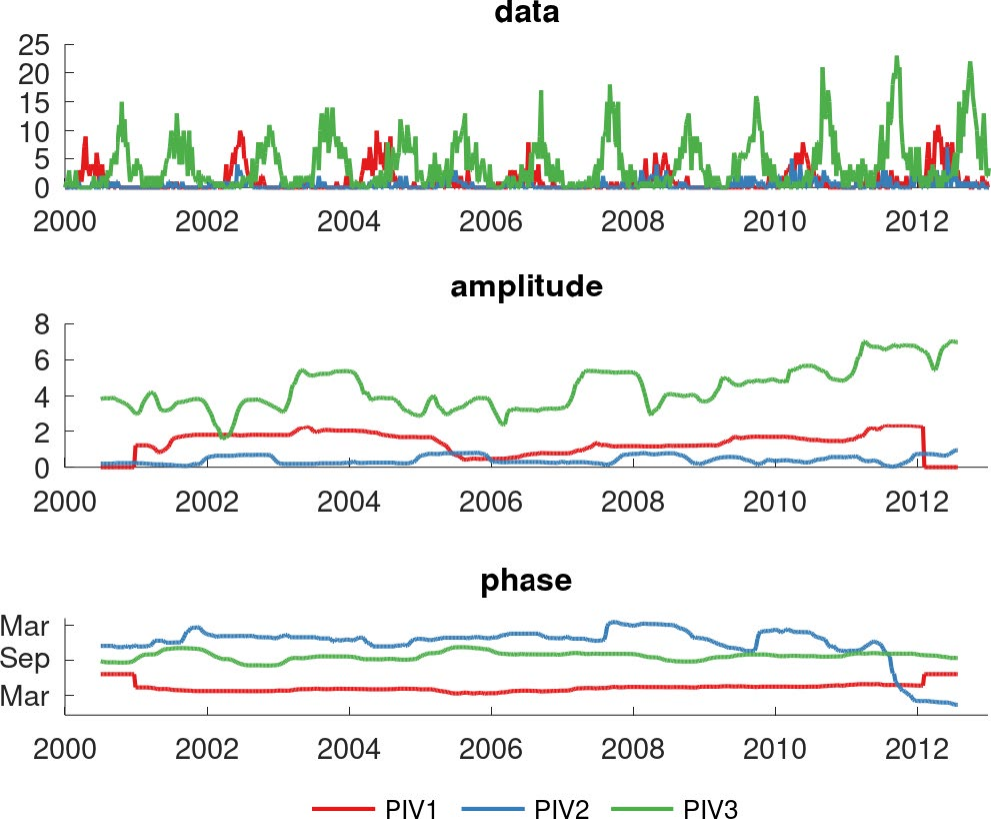
Analysis of seasonality of the three PIV serotypes. The top figure presents weekly incidence of each serotype; the middle figure presents the amplitude of seasonal epidemics, representing the peak size; the bottom figure shows the phase or timing of the peak in the seasonal epidemics

Hospitalised PIV detections by serotype had a distinct seasonal pattern: PIV‐1 peaked in the autumn months (April), PIV‐2 in the winter (June) and PIV‐3 in the spring (September; Figure 3). This was supported by the complex demodulation analysis of all PIV positive detections regardless of hospitalisation (Figure 4), which shows both the amplitude (size of epidemic peak; middle plot) and the phase (timing of epidemic peak; bottom plot) for each serotype. The highest incidence was seen in PIV‐3 (middle plot), with some indication of increasing levels towards the end of the study period. The phase plot indicates PIV‐1 peaked consistently in March/April, with PIV‐3 commonly peaking around September but with some variation by year. PIV‐2 had the lowest incidence and least consistency in peak timing.

Estimates from the mathematical modelling analysis are shown in Table 2, with plots of the model fit to data in Figures S1‐S3. The model successfully captured the timing of the epidemic peaks and showed biennial behaviour for PIV‐3, although low numbers of cases impaired the fit to PIV‐2. For the better fitting PIV‐1 and PIV‐3 models, reproduction numbers (*R*_0_) of 1.24 (PIV‐1) and 1.71 (PIV‐3) were estimated. The *R*_0_ for PIV‐2 was estimated at 2.2. PIV‐3 had the greatest seasonality, and PIV immunity duration was around 300‐550 days, depending on serotype (Table 2).

**TABLE 2:** Estimates of the reproduction number (*R*_0_), the strength of seasonality, the duration of infectivity and the duration of immunity for the three PIV strains as fitted to an SEIRS compartmental model

	*R*_0_	% seasonality	Infectivity (d)	Immunity (d)
PIV‐1	1.24	6.1	3.3	476
PIV‐2	2.23	7.7	12.9	303
PIV‐3	1.72	12.8	6.4	556

## 4 DISCUSSION

We used a population‐based dataset to describe the age‐specific incidence and seasonal dynamics of PIV in children. We found that overall PIV rates were highest in children aged less than 2 years, particularly in infants aged 1‐5 months, and that PIV‐3 had the highest incidence of all examined serotypes for each age group, followed by PIV‐1, then PIV‐2. Rates of PIV‐confirmed hospitalisation were higher in Aboriginal children than non‐Aboriginal children. PIV‐3 peaked in the spring months around September, while PIV‐1 peaked biennially in the autumn months around April, and PIV‐2 followed a similar biennial pattern, but tended to peak in the winter months, around June. Finally, seasonal viral testing peaks, driven by RSV and flu activity, did not coincide with overall PIV‐1 and PIV‐3 activity peaks, suggesting a degree of under‐ascertainment due to emergent seasonal testing patterns.

Previous studies have investigated the burden of PIVs, and many population‐level studies generally report a high prevalence of PIV serotypes in children aged under 5 years.^1^, ^7^, ^12^ Studies of age‐specific PIV rates in young children are rarer, but usually report PIV rates to be higher in children aged less than 2 years, peaking sometime in the first 6 to 12 months of life.^3^, ^13^, ^30^, ^31^ Similarly, we attribute the highest burden of PIV‐associated hospitalisations to children under 1 year old, with PIV rates peaking in children aged 1 to 5 months. Interestingly, this age bracket is consistent with an anti‐PIV maternal antibody half‐life of 53 days reported by previous investigations, suggesting that maternal antibodies may not be universally protective against PIV infection for the first months of life.^32^

Global studies suggest that, of all serotypes, PIV‐3 presents the most immediate public health concern for children under five, being the most frequent cause of morbidity, followed by PIV‐1, then PIV‐2.^3^, ^7^, ^12^, ^17^ Compared to influenza virus and RSV assessments from this same dataset,^1^ we have shown, for children aged 1‐5 months, a higher hospitalisation rate for PIV‐3 alone than for influenza hospitalisations, and PIV‐3 rates eclipsed influenza rates in children until the age of 2. Similarly, previous studies variously attribute a disease burden of PIVs comparable to, or greater than, influenza viruses, yet significantly lower than RSV.^2^, ^19^, ^31^ The burden of all PIV serotypes was significantly higher in Aboriginal children, compared to non‐Aboriginal children. This result has been reported for other respiratory viruses in Indigenous populations from around the world, in part due to increased risk factor exposure in marginalised, often rural, population groups.^33^, ^34^

Aside from PIV‐2, our estimates of *R*_0_ and duration of immunity for PIV were consistent with those of other respiratory pathogens.^35^, ^36^ Owing to the low sample size for PIV‐2, our ability to fit a seasonal model to that serotype was limited. Though our observation of biennial PIV‐1 circulation resembles that made by previous studies, Western Australian PIV‐1 peaks were found to occur in even‐numbered years, rather than the odd‐numbered year peaks frequently reported in northern hemisphere studies.^7^, ^12^, ^15^ This biennial peaking pattern for PIV‐1 is consistent with that observed for RSV,^37^, ^38^ which may result from a degree of residual immunity in off years, but it is unclear why this is not seen in PIV‐3. Though PIV‐3 was present in circulation each year, PIV‐3 frequency was slightly lower in even‐numbered years when PIV‐1 was in circulation, perhaps indicating a degree of competitive inhibition between PIV serotypes.^39^ These factors may also play a role in the discrepancy in PIV‐1 peak years between northern and southern hemisphere sites. Despite the low number of PIV‐2 detections over the study period, hindering our ability to fit a mathematical model to PIV‐2, we were able to detect annual winter PIV‐2 activity. Though most studies generally agree with the late‐autumn‐to‐winter timing of PIV‐2 activity in temperate regions, there are conflicting international reports of annual, biennial and biannual PIV‐2 peaks across climate regions.^7^, ^12^, ^15^, ^18^ Indeed, respiratory viral seasonality is expected to vary by climate; previous studies have observed differences in RSV and influenza seasonality between temperate and tropical climate regions.^40^, ^41^ Similar variations in PIV activity between climatic regions seem likely, perhaps accounting for observed discrepancies in PIV‐2 seasonality; future studies with greater sample sizes from tropical areas will aid in understanding the relationship between PIV seasonality and climate. Finally, through our seasonality modelling approach, a continuous upward trend in PIV‐3 activity was noted for the final 3 years of the study period, which may warrant further local monitoring.

As we have previously reported from this dataset, respiratory viral testing only occurred in 48% of hospitalisations for respiratory infections, and 9% of total hospitalisations.^1^ Therefore, our PIV‐confirmed hospitalisation rates are the minimum estimates of the true burden of paediatric PIV in WA. Despite this limitation, linkage of hospital administrative records with laboratory data is a valuable source of data for disease burden studies, due to the broad clinical presentation of PIV infections and inferior sensitivity and specificity of diagnostic code‐based epidemiology.^42^

## 5 CONCLUSION

In summary, we have shown that PIVs present a significant health burden in Australian children, with PIV‐3 the largest contributor to that burden. PIVs exhibited distinct seasonality patterns, which, for PIV‐1 and 3, rarely overlapped. The burden posed by PIV‐2 appears to be much lower, and the seasonality of PIV‐2 remains uncertain. To better understand the need for therapeutic and vaccination options, as well as public health response, continued surveillance of all serotypes is recommended in both resource‐rich and poor settings. Given the burden of disease, interventions targeting serotypes 3 and 1 should be prioritised for children in the first 6‐12 months of life, recognising the differing seasonality of PIV serotypes. In the absence of a PIV vaccine, further work is needed to understand and identify potentially modifiable risk factors for PIV in Aboriginal and non‐Aboriginal children to reduce the PIV health burden.

## CONFLICT OF INTEREST

The authors declare no conflicts of interest.

## AUTHOR CONTRIBUTION

**Daniel Rene Lovell Greiff:** Conceptualization (equal); Formal analysis (lead); Investigation (lead); Methodology (lead); Project administration (lead); Software (equal); Validation (supporting); Visualization (lead); Writing‐original draft (lead); Writing‐review & editing (lead). **Alice Patterson‐Robert:** Conceptualization (equal); Formal analysis (lead); Investigation (lead); Methodology (lead); Software (supporting); Visualization (equal); Writing‐review & editing (supporting). **Kathryn Glass:** Conceptualization (equal); Formal analysis (equal); Investigation (equal); Methodology (supporting); Software (equal); Supervision (lead); Validation (lead); Visualization (equal); Writing‐review & editing (equal). **Christopher Blyth:** Conceptualization (supporting); Funding acquisition (equal); Supervision (supporting); Validation (equal); Writing‐review & editing (equal). **Hannah C Moore:** Conceptualization (equal); Data curation (lead); Formal analysis (supporting); Funding acquisition (lead); Investigation (supporting); Methodology (supporting); Project administration (supporting); Resources (lead); Software (equal); Supervision (lead); Validation (lead); Writing‐review & editing (equal).

### PEER REVIEW

The peer review history for this article is available at https://publons.com/publon/10.1111/irv.12838.

## Supporting information

Supplementary Material

## Data Availability

Datasets used in this study are available, by application, via the corresponding author, from the WA Data Linkage Branch in compliance with ethical considerations.
