# Supplementary material for: Epidemiology and seasonality of human parainfluenza serotypes 1‐3 in Australian children
Source: Influenza Other Respir Viruses. 2021 Jan 24;15(5):661–9. doi: 10.1111/irv.12838 (PMC8404051; doi:10.1111/irv.12838)
Supplement: Supplementary file 1 — Supplementary Material [file IRV-15-661-s001.docx]

# Supplementary Figures

**Supplementary Table 1:** Parameters used in seasonality model.

| **Parameter Description** | | **Parameter Value** |
| --- | --- | --- |
| μ | birth and death rate | 0.00005 |
| β_0_ | median transmission rate | fitted |
| β_1_ | strength of seasonality | fitted |
| φ | time shift to determine peak transmission rate | fitted |
| β(t) | seasonally forced transmission rate | β_0_ (1+ β_1_sin(2π(t+ φ)) |
| ω | duration of immunity | fitted |
| γ | duration of infectiousness | fitted |
| σ | latent period | 0.3846 |
| N | population size | 100,000 |

**Supplementary table 2. Frequency of detection and incidence rates of PIV-1-3 in Aboriginal and non-Aboriginal children, Western Australia, 2000-2012.** All rates presented by age strata, per 100,000 person-years. Numbers less than or equal to five are suppressed in this figure, out of concern for patient privacy.

| **PIV Type** | **Age Group** | **Aboriginal** | | **Non-Aboriginal** | | **Incidence Rate Ratio (95% CI)** |
| --- | --- | --- | --- | --- | --- | --- |
|  |  | N | Rate (95% CI) | N | Rate (95% CI) |  |
| **PIV-1** | <1 month | <5 | 98.5 (24.6, 394.0) | 10 | 35.2 (18.9, 65.4) | 2.80 (0.30, 13.14) |
|  | 1-5 months | 21 | 207.2 (135.0, 318.0) | 74 | 52.2 (41.6, 65.6) | 3.97 (2.32, 6.51) |
|  | 6-11 months | 20 | 165.8 (107.0, 257.0) | 64 | 38.1 (29.8, 48.6) | 4.35 (2.49, 7.29) |
|  | 12-23 months | 19 | 79.3 (50.7, 124.0) | 119 | 35.9 (30.0, 43.0) | 2.21 (1.28, 3.60) |
|  | 2-3 years | 8 | 17.0 (8.5, 34.0) | 86 | 13.3 (10.8, 16.5) | 1.28 (0.53, 2.63) |
|  | 4-16 years | 9 | 6.2 (3.2, 11.9) | 55 | 2.8 (2.1, 3.4) | 2.26 (0.98, 4.60) |
| **PIV-2** | <1 month | 0 | 0 | <5 | 14.1 (5.3, 37.5) | *Not Applicable* |
|  | 1-5 months | <5 | 49.3 (20.5, 118.0) | 35 | 24.7 (17.7, 34.4) | 2.00 (0.61, 5.12) |
|  | 6-11 months | 11 | 91.1 (50.5, 165.0) | 32 | 19.0 (13.5, 26.9) | 4.79 (2.18, 9.75) |
|  | 12-23 months | 7 | 29.2 (13.9, 61.3) | 27 | 8.2 (5.6, 11.9) | 3.58 (1.32, 8.44) |
|  | 2-3 years | <5 | 6.39 (2.1, 19.8) | 13 | 2.0 (1.2, 3.5) | 3.17 (0.58, 11.54) |
|  | 4-16 years | 8 | 5.5 (2.7, 11.0) | 38 | 1.9 (1.4, 2.6) | 2.90 (1.17, 6.32) |
| **PIV-3** | <1 month | <5 | 197.1 (74.0, 525.0) | 52 | 182.9 (139, 239.7) | 1.08 (0.28, 2.93) |
|  | 1-5 months | 84 | 828.8 (668.9, 1000) | 305 | 215.2 (192.0, 241.0) | 3.85 (2.99, 4.92) |
|  | 6-11 months | 77 | 637.9 (510.2, 798.4) | 274 | 163.0 (144.8, 182.9) | 3.91 (3.00, 5.06) |
|  | 12-23 months | 63 | 262.9 (205.1, 335.9) | 331 | 99.9 (89.7, 111.0) | 2.63 (1.98, 3.45) |
|  | 2-3 years | 30 | 63.9 (44.7, 91.4) | 197 | 30.5 (26.6, 35.1) | 2.09 (1.37, 3.08) |
|  | 4-16 years | 17 | 11.7 (7.30, 18.9) | 114 | 5.7 (4.8, 6.9) | 2.06 (1.16, 3.44) |
|  | **Total** | <388 | 162 (146, 179) | <1,830 | 55.3 (52.8, 57.9) | 2.93 (2.62, 3.27) |


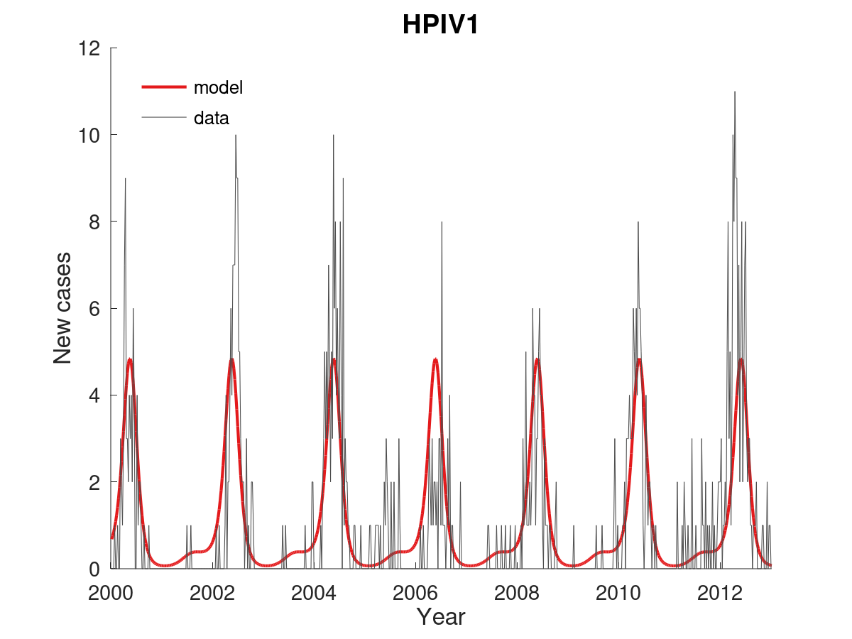


**Figure S1:** PIV1 data (grey) and model fit to these data.


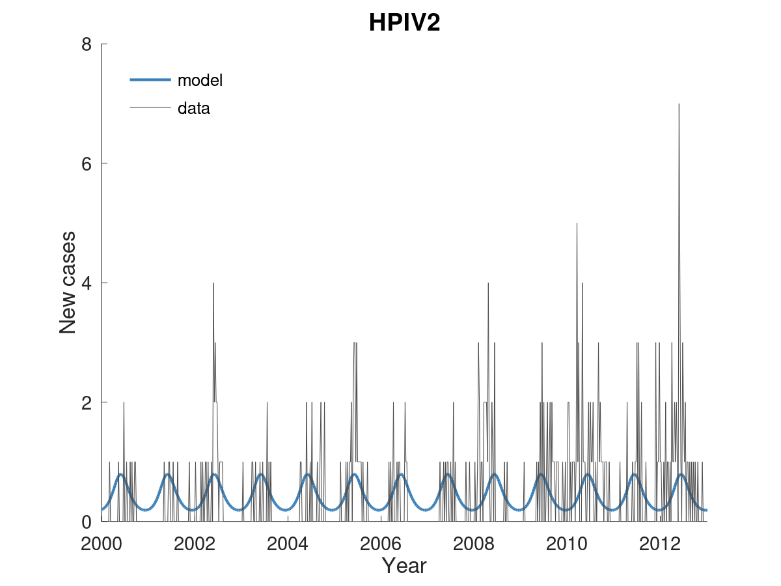


**Figure S2:** PIV2 data (grey) and model fit to these data (blue).


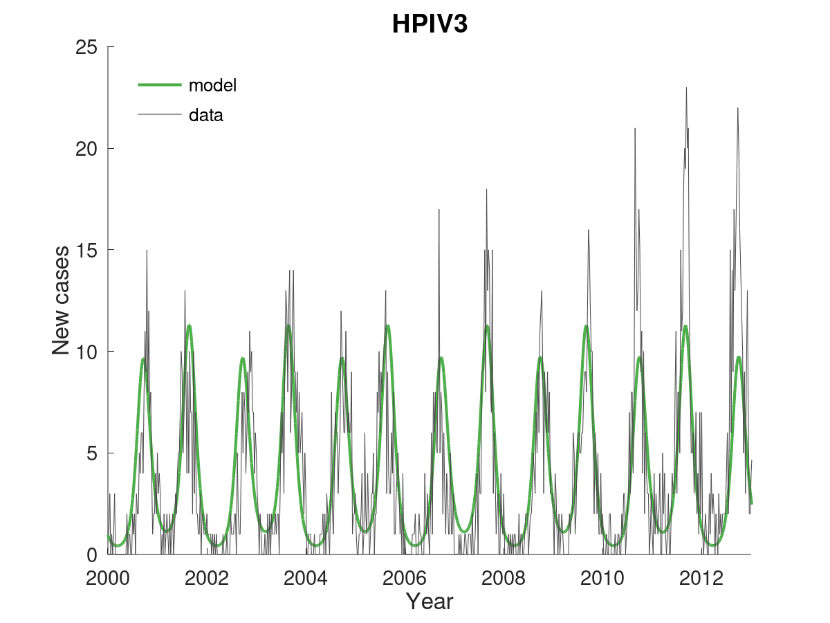


**Figure S3:** PIV3 data (grey) and model fit to these data (green).
